# Accurate Lattice Free Energies of Packing Polymorphs from Probabilistic Generative Models

**DOI:** 10.1021/acs.jctc.4c01612

**Published:** 2025-02-21

**Authors:** Edgar Olehnovics, Yifei Michelle Liu, Nada Mehio, Ahmad Y. Sheikh, Michael R. Shirts, Matteo Salvalaglio

**Affiliations:** † Thomas Young Centre and Department of Chemical Engineering, University College London, London WC1E 7JE, United Kingdom; ‡ Molecular Profiling and Drug Delivery, Research & Development, AbbVie Bioresearch Center, Worcester, Massachusetts 01605, United States; § Molecular Profiling and Drug Delivery, Research & Development, AbbVie Inc., North Chicago, Illinois 60064, United States; ∥ University of Colorado Boulder, Boulder, Colorado 80309, United States

## Abstract

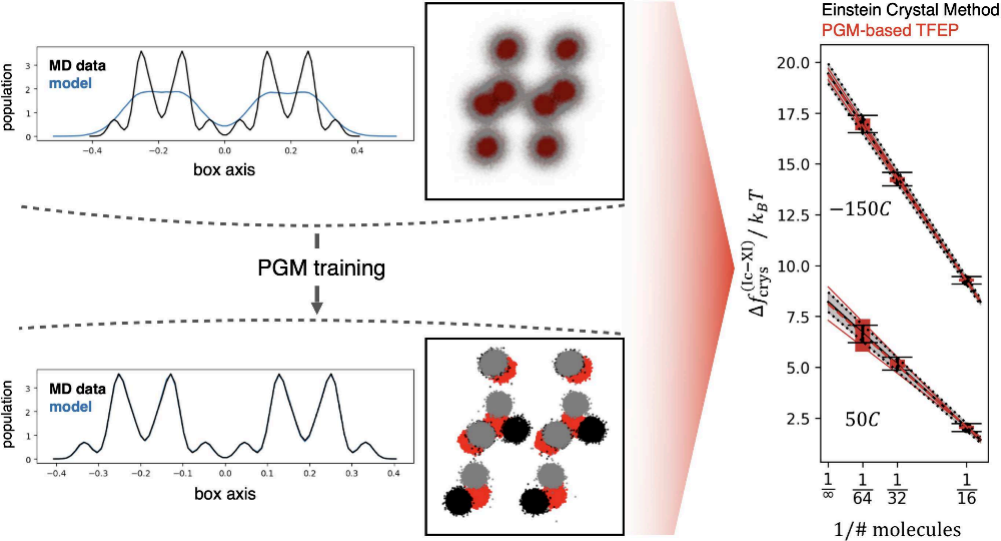

Finite-temperature lattice free energy differences between polymorphs of molecular crystals are fundamental to understanding and predicting the relative stability relationships underpinning polymorphism, yet are computationally expensive to obtain. Here, we implement and critically assess machine-learning-enabled targeted free energy calculations derived from flow-based generative models to compute the free energy difference between two ice crystal polymorphs (Ice XI and Ic), modeled with a fully flexible empirical classical force field. We demonstrate that even when remapping from an analytical reference distribution, such methods enable a cost-effective and accurate calculation of free energy differences between disconnected metastable ensembles when trained on locally ergodic data sampled exclusively from the ensembles of interest. Unlike classical free energy perturbation methods, such as the Einstein crystal method, the targeted approach analyzed in this work requires no additional sampling of intermediate perturbed Hamiltonians, offering significant computational savings. To systematically assess the accuracy of the method, we monitored the convergence of free energy estimates during training by implementing an overfitting-aware weighted averaging strategy. By comparing our results with ground-truth free energy differences computed with the Einstein crystal method, we assess the accuracy and efficiency of two different model architectures, employing two different representations of the supercell degrees of freedom (Cartesian vs quaternion-based). We conduct our assessment by comparing free energy differences between crystal supercells of different sizes and temperatures and assessing the accuracy in extrapolating lattice free energies to the thermodynamic limit. While at low temperatures and in small system sizes, the models perform with similar accuracy. We note that for larger systems and high temperatures, the choice of representation is key to obtaining generalizable results of quality comparable to that obtained from the Einstein crystal method. We believe this work to be a stepping stone toward efficient free energy calculations in larger, more complex molecular crystals.

## I Introduction

Free energy estimates are key to quantitatively understanding physical-chemical processes ranging from solubility to binding affinity.^1−4^ In the case of polymorphism of molecular crystals in particular, accurate and efficient lattice free energies are needed to determine the relative stability of crystal packings, which is important in many industries, including chemicals, pharmaceuticals, semiconductors, and food products.^5−9^ An accurate prediction of the relative thermodynamic stability of polymorphs at different temperatures requires calculating the entropic contribution to free energy. Including such thermodynamic detail is often prohibitively expensive due to the anharmonic nature and the presence of low-frequency modes in the vibrational free energy.^8,10−15^ For free energy calculations to be of industrial relevance, free energy methods are needed that are suitably cheap and scalable for deployment on larger data sets of putative finite temperature polymorphs while retaining accuracy levels on par with states of the art classical anharmonic free energy methods.^8,12,13^ Given an atomistic model of the system of interest and a putative crystal structure, its finite-temperature lattice free energy can be rigorously computed using statistical mechanics computational techniques, often based on the principles of free energy perturbation and thermodynamic integration.^16,17^ Several approaches have been proposed in the literature, including various implementations of the Einstein crystal method,^17−19^ diabat approaches drawing inspiration from lattice-switch Monte Carlo and Marcus theory^20−22^ and enhanced sampling methods based on introducing biasing potentials,^23−25^ to name a few. While these approaches are theoretically well established and formally yield fully anharmonic lattice-free energies, their computational cost is frequently too demanding for large-scale deployment. For this reason, the current state of the field relies on the ad hoc application of exact methods such as the Einstein crystal and the more widespread use of methods based on the harmonic approximation, which are nowadays routinely deployed to carry out a final refinement of zero-Kelvin energy landscapes.^6,12^

One of the main limitations of formally exact methods based on statistical mechanics is that they typically require a significant amount of computing effort to sample intermediate, unphysical states, which increases the computational cost without providing inherent physical insights.^16,17^ Taking the Einstein crystal method as the “gold standard” example of a physics-based, formally exact approach for calculating lattice-free energies, we note that its convergence requires devising a chain of states with overlapping configurational distributions that connect the Hamiltonian describing the physical crystal polymorph to the Einstein crystal Hamiltonian. To obtain convergence, neighboring Boltzmann distributions along such a chain of states must sample overlapping configurations, and in each state, configurations must be sampled ergodically. Thus, sampling sets of tens to hundreds of perturbed Hamiltonians are typically required to achieve well-converged free energy estimates for a single putative crystal form.

The emergence of machine learning methods capable of training deterministic bijective coordinate transformations that directly map between the probability distributions of two arbitrarily different metastable states of interest, in this case, the polymorphic cell of interest and the Einstein crystal reference state offers a promising alternative to the costly direct sampling of intermediate, unphysical states.^26−31^ We have recently shown that such approaches, coupled with BAR^32^ and MBAR^33^ reweighting techniques for free energy estimation can yield very accurate free energy differences between conformational states of isolated molecules.^34−39^

Here, we take stock of the lessons learned in ref^39^ to critically compare two different methods of representing the configurations of flexible molecules in a crystal for training invertible flow-based probabilistic generative models (PGMs) that can effectively map between two metastable water ice crystal structures (Ice XI and Ic), modeled with an empirical classical force field. In particular, we assess the efficiency and accuracy of the lattice-free energy estimates obtained by ML-enabled targeted free energy estimation against ground truth estimates obtained with an Einstein Crystal method. We examine the effect of different temperatures and system sizes on the convergence of free energy estimates and the quality of extrapolating these results to the thermodynamic limit. We note that the two temperatures examined (−150 and 50 °C) are meant to stress test the methods. For instance, at higher temperatures, we can expect the configurational distributions to be significantly less localized and more anharmonic, making them more challenging to model using neural networks.

## II Probabilistic Generative Models for Lattice Free Energy Calculation

To quantitatively estimate lattice free energies, we build and expand on our earlier work,^39^ where we identified several useful heuristics for applying reweighted PGMs to obtain accurate free energy estimates. The only MD data that is required for training and reweighting the PGMs is that which is sampled in the physical macrostates of interest. This approach, therefore, entirely avoids sampling multiple intermediate Hamiltonians (details provided in the *Simulations Setup* section), typical of free energy perturbation methods such as the Einstein Crystal.^39,43^ In ref,^39^ we used isolated small molecules in a vacuum as representative case studies, with 3*N* – 9 out of 3*N* – 6 of the relevant intramolecular degrees of freedom (DOFs) represented using internal coordinates.^44^ Those PGMs relied on a *mixed coordinate* representation of the given molecule.^34^ Specifically, we kept three of the atoms represented in Cartesian coordinates to serve as a starting point for a bijective reconstruction of all remaining atoms of the molecule from the internal coordinates. We referred to these three Cartesian atoms as the *Cartesian block*. When dealing with isolated molecules in a vacuum, the Cartesian block only has three relevant DOFs. However, in systems containing more than one molecule, like crystal supercells, all nine DOFs of the Cartesian block of each molecule must be jointly modeled by the PGM.

**Figure 1:**
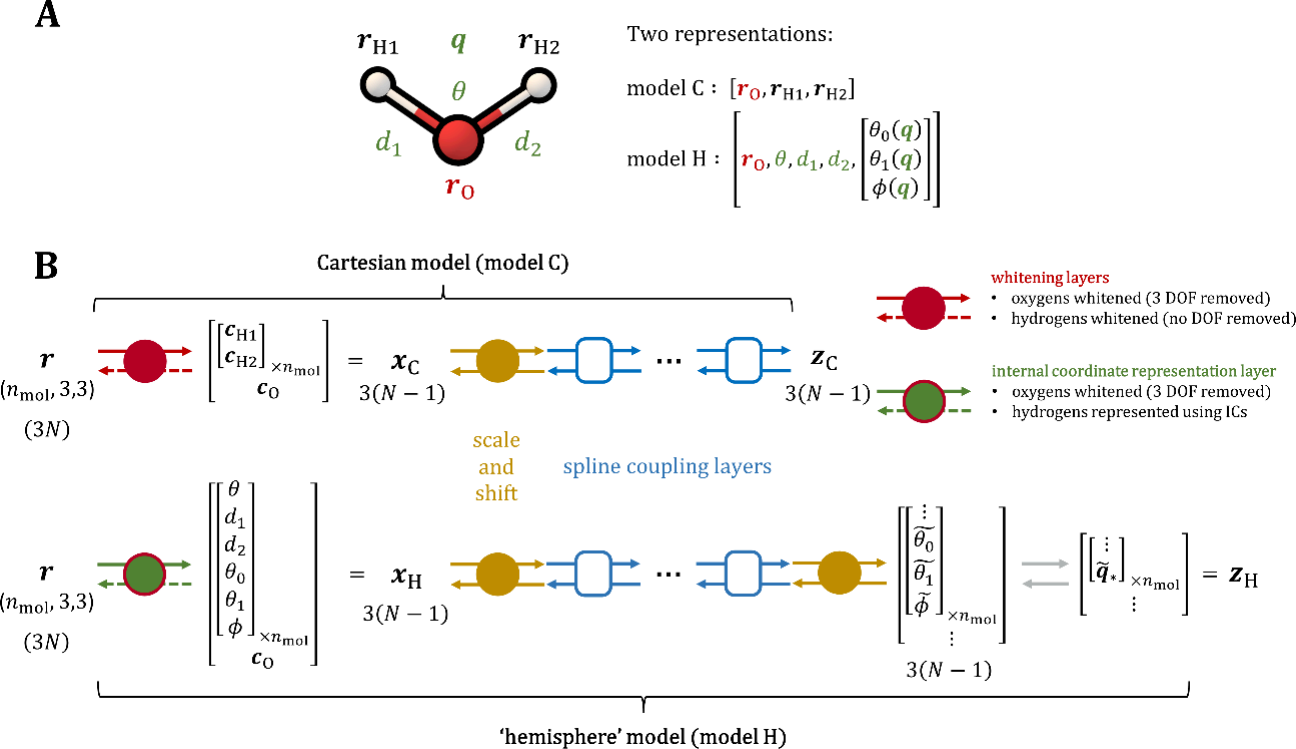
(A) Illustration of a water molecule with two representations (model C and model H) of the 9 DOFs. (B) Illustration of the two model architectures compared in this work (model **C**; top and model **H**; bottom). Representations: Both models start by transforming the Cartesian coordinates of the system ***r***, containing *N* atoms, as discussed in the main text, to create a vector ***x*** containing 3(*N* – 1) marginal variables. These variables are then rescaled to a fixed model range (e.g., [−1, 1] or [−1, 1), depending on the type of variable). Mapping: Both models used one-dimensional monotonically increasing rational quadratic splines^40^ (with five trainable knots) to transform ***x*** in an elementwise manner according to coupled flow architecture.^41^ This was implemented as in ref.^39^ In each system, 4 coupling layers were used in the case of the Hemisphere model, and a maximum number of coupling layers was used in the Cartesian model. The partitioning of variables in each layer was based on the heuristic discussed in SI Figure S1.^42^ The coupling layers invertibly transform ***x*** to ***z***. The maps were trained to approach a uniform remapped distribution in ***z***. For this reason, in model **H**, the variables associated with quaternions are transformed back to 4D unit vectors on the specified hemisphere. The details are discussed in the main text. The amount of data used for training and validating the PGMs was 200,000 samples (extracted from a 20 ns trajectory) in each system containing 16 or 32 molecules and 400,000 samples (extracted from a 40 ns trajectory) in each system with 64 molecules. A training:validation split of 3:1 was used during each training run.

Here, we use flow-based PGMs to compute the Helmholtz FE differences between crystal supercells using two ice polymorphs (Ic and XI) as simple but representative examples of systems requiring the explicit representation of all nine DOFs for all Cartesian blocks. For such systems, it is important to consider the most cost-effective way of representing a Cartesian block inside the PGM. In this regard, we highlight ref,^30^ which has contributed a powerful method of representing the Cartesian block based on a unit-quaternion to describe the rotational state of a given molecule. In particular, they have demonstrated that building a normalizing flow model with this representation allows learning an accurate approximation of the Boltzmann distribution over DOFs belonging to atoms that are not covalently bonded and, thus, obtaining FE estimates of ice XI crystal supercells modeled with rigid water molecules.^30^ In the following, we compare two PGM architectures, respectively implementing a quaternions-based representation inspired by ref^30^ and a direct Cartesian representation. These two models only differ in how the hydrogen atoms in each water molecule are represented. The first representation is based directly on their Cartesian coordinates (model **C**, illustrated in Figure 1A), while the second relies on unit-quaternions and internal coordinates (model **H**, illustrated in Figure 1B). Details of the models are discussed in sections IIA and IIB, respectively. To assess their accuracy, efficiency, and ability to deal with an increasing number of DOFs, we extended our comparison to three system sizes and two temperatures.

To maintain consistency in comparing free energy differences computed from the two models and the ground truth, we established the following ground rules:

- Both types of models were trained and evaluated on the same data, following the same protocol. For instance, the training validation split was fixed to 3:1 in all cases. This MD data was taken from λ = 1 simulations defined in section IIH to establish a fair comparison with the Einstein crystal method. Each model was trained and evaluated on batches of 1,000 and 10,000 configurations, respectively.
- The three DOFs defining the position of the center of mass were removed from both types of flows identically by *whitening* the Cartesian coordinates of all oxygen atoms, as discussed in section IIC.
- Each spline coupling layer (represented in blue in Figure 1) used a specific nonrandom conditioning protocol (displayed in Figure S1). The number of trainable parameters was adjusted by varying the number of coupling layers to minimize the variance of FE estimates obtained using the BAR_V estimator (defined in section IID) evaluated on the models during training. Models were trained for as long as necessary to observe a convergent behavior in the running average of these estimates, as defined in section IIE.

Moreover, we note that it was possible to construct the trainable part of both models using standard spline coupling layers, analogously to our previous work in isolated molecules.^39^ As such, both models (**C** and **H**) used standard coupling layers^39^ that are not inherently equivariant to permutations of any DOFs. It was possible to use this approach because none of the water molecules in the supercells we compared swapped lattice sites within the MD-accessible time scales. We note that PGM architectures can be equipped with equivariant functionality by replacing the standard multilayered perceptrons with attention-based coupling mechanisms.^26−28,30,31^ The following sections describe in detail the approach adopted in models **C** and **H**, respectively.

### A Cartesian Representation of Hydrogen Atoms

In the Cartesian model (model **C** in Figure 1B), the positions of the hydrogen atoms in the whole system [***r***_H1_, ***r***_H2_] were represented directly with their Cartesian coordinates. Meanwhile, the oxygen atoms in the system (***r***_O_) were instead represented using whitened Cartesian coordinates, as described in section IIC. Taken together, the variable ***x***_C_ shown in Figure 1, describes all the relevant *N*(*N* – 1) marginal DOFs of the ice crystal. Finally, the shift and scale layer (yellow in Figure 1) was initialized to fit the one-dimensional marginal variables of ***x***_C_ into the working interval of the 1D splines. This step takes advantage of the minimum and maximum values explored by each 1D marginal variable in the MD training and validation data to define the one-dimensional linear transformations to carry out the scale and shift, exactly like in ref.^39^ Likewise, the spline coupling layers in this work were also implemented according to methods detailed in ref,^39^ with each marginal variable of model **C** assigned to a nonperiodic [−1, 1] interval. The uniform log base distribution (ln *p*_0_) in model **C** was therefore set to^39^
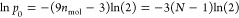
1

### B Hemisphere Representation of Hydrogen Atoms

In the hemisphere model (model **H** in Figure 1B), the positions of the hydrogen atoms were described with the help of unit quaternions. The following paragraphs describe how this representation was implemented.

Reference^30^ shows that Cartesian coordinates of the two hydrogen atoms (***r***_H1_ and ***r***_H2_) of a water molecule ***r***_mol_ = [***r***_O_, ***r***_H1_, ***r***_H2_] can be efficiently represented by a set of independent internal coordinates (***x***_mol_), consisting of two bond lengths (*d*_1_, *d*_2_), one bond angle (θ), and one (flip invariant) unit-quaternion , as well as ***r***_O_.^30,45^ For brevity, only the inverse of this transformation is discussed here. The configuration of a water molecule can be reconstructed from these coordinates using the following equation, where we adopt the approach detailed in ref^46^ to transform a unit-quaternion into a rotation matrix ***R***(***q***):^30,45,46^
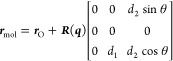
2

In relation to eq 2, refs^30,45^ provide the following closed-form expression of the log-volume change:
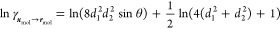
3

We observe that eq 3 gives the same quantities as a , with eigenvalues λ_1_, ..., λ_9_ obtained from diagonalizing ***J***^*T*^***J***, where  is the Jacobian of eq 2 given by automatic differentiation. eq 3 was therefore evaluated when reconstructing a water molecule (i.e., mapping ***x***_mol_ → ***r***_mol_). When mapping in the opposite direction (i.e., ***r***_mol_ → ***x***_mol_), as defined in ref,^30^ the log volume change of  was used.^30,45^

In model **H**, all of the intramolecular DOFs (i.e., the bond lengths and angles , see Figure 1) were directly concatenated to the whitened Cartesian coordinates of the oxygen atoms (***c***_O_), with the latter being treated in the same way as in model **C**.

The remaining 3*n*_mol_ DOFs of the system, corresponding to the *n*_mol_ unit-quaternions (specifying the rotations of the molecule in a supercell relative to a fixed reference frame ensured by the nonrotating supercell vectors), were treated as discussed below.

Each unit-quaternion, representing the rotation of a given molecule in an arbitrary crystal data set, may exist anywhere on the *S*^3^ hyper-sphere. Thus, its four marginal coordinates [*q*_0_, *q*_1_, *q*_2_, *q*_3_] are coupled by the unit-length constraint. Moreover, antipodal quaternions represent equivalent rotation matrices (i.e., ***R***(***q***) = ***R***(−***q***)). This means that any hemisphere of *S*^3^ is sufficient to describe all possible rotations, or equivalently, only half of *S*^3^ is sufficient to specify a set of all unique quaternion-based representations of all 3D rotations.^47,48^ When considering the curvature of *S*^3^, or its hemisphere, it is clear that an explicit global parametrization of this space is impossible without encountering singularities. Instead, the recommended singularity-free approach is to work directly with unit vectors embedded in  (implicit representation).^46^ Indeed, this is exactly how ref^30^ approached the problem of mapping between distributions of rotations. Specifically, two novel flip-equivariant bijectors (*symmetrized Moebius transformations* and *symmetrized projective convex gradient maps*) were developed in ref^30^ that enable modeling smooth flip-symmetric flows on the surface of any flip-symmetric sphere. In the case of *S*^3^, these bijectors serve as robust flow architectures for modeling distributions on the rotation manifold (SO3).^30^ Alternative ways of learning normalized distributions of rotations that do not rely on flows can also be found in the literature and retain the unit-vector representation.^49,50^

All quaternions are assigned to a specific hemisphere and then globally parametrized using standard hyper-spherical coordinates despite the presence of singularities. This choice was implemented to provide model **H** with sufficient representational power while minimizing computational costs.

The hemisphere was specified by q_*_ = sign(*q*_0_)***q***, where , and the associated maps are
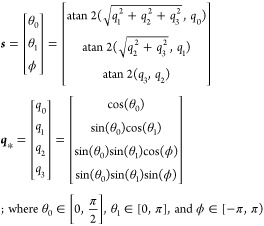
4

The log-volume change of eq 4, in the ***s*** → q_*_ direction is ln(sin^2^(θ_0_) sin(θ_1_)). When transforming in the opposite direction, the same expression is multiplied by −1. The singularities in this quantity are considered at points where sin(*x*) = 0 for any *x*, where the volume, and thus density, cannot be defined. For numerical stability of dealing with samples flowing near these points, the arguments of the log were *clipped* to be greater than a small but finite threshold (1e-8). Generally, clipping of this type can introduce errors during inference, which in the flow-based PGMs can be quantified by a mismatch in log volume changes associated with the forward and inverse transformations of the data. For this reason, we systematically checked the inversion accuracy of model **H** in both directions, before and after training, to ensure that the approximation introduced in the chosen representation is within reasonable tolerance levels. An example of this analysis is discussed in SI Figure S7. One can assume that the small patches of configurational space surrounding the singularities in each molecular configuration are negligible compared to the total volume of configurational space. Thus, the errors associated with samples flowing near these regions may, on average, yield a negligible contribution during reweighting.

Furthermore, since ***s*** does not preserve the antipodal flip-symmetry at the edge of the hemisphere, we note that in the systems we examined so far it was possible to rotate the global reference frame of the supercell data such that, for each molecule in the data set, the marginal distribution in θ_0_ is shifted away from both 0 (one of the singularities) and  (edge of the hemisphere). More generally, one can also note that in a stable crystal system where none of the molecules samples significantly larger rotational angles, and thus their quaternion distribution on *S*^3^ is quite narrow, one can define an arbitrary per-molecule rotation (i.e., a translation of the MD data set on *S*^3^) such that each molecule maps to a formally well behaved joint distribution in the hyper-spherical coordinates (***s***). In this regime, the three marginal variables of ***s*** can be transformed and treated independently.

That being said, we intentionally did not limit the model **H** from being capable of generating all possible rotations, even though this constraint can be trivially imposed on the marginals of ***s***, as part of scaling and shifting layer.

After each unit-quaternion is transformed to ***s***, the combined set of 3*n*_mol_ rotational DOFs of the crystal were concatenated to the rest of the marginal variables mentioned earlier to give ***x***_H_ shown in Figure 1B.

Akin to model **C**, the coupling layers in a model **H** were trained to flatten the configurational distribution of the MD data, to map it as close as possible to a uniform distribution over ***z***_H_ (Figure 1). In terms of achieving a uniform distribution of rotations, this corresponds to uniformly sampling the surface of the hemisphere of *S*^3^. For this reason model **H** transforms ***s*** back to q_*_ on both sides of the flow, including the *noisy* side (Figure 1). The log base distribution ln *p*_0_ in model **H** was therefore set to
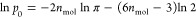
5

The first term in eq 5 originates from the fact that a hemisphere of *S*^3^ has a surface area of π^2^. The second term takes into account the 6 remaining nonrotational DOFs in each molecule, with 3 global center-of-mass degrees of freedom of the supercell missing from the flow.

### C Virtual Atoms and Center of Mass Treatment

The classical force field used to model water molecules in the polymorphic forms of ice investigated was TIP4P/Ice: a four-body potential that, alongside the oxygen and two hydrogen atoms, employs an additional virtual atom (***r***_M_). The dynamics of the virtual atom do not contribute any additional DOF to the system because its position can be reconstructed without uncertainty as
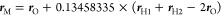
6where ***r***_*i*≠*M*_ represents the position of atom *i*. As such, the virtual atoms were removed before feeding molecular coordinates to any PGM model. When sampling the supercell configurations of ice polymorphs using the model, virtual atoms were reconstructed using eq 6.

The center of mass (COM) of the oxygen atoms was removed from all the MD-generated configurations used in the training or validation of the PGM models as
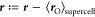
7

After applying eq 7, in which the periodic boundary conditions (PBCs) of the supercell box are not taken into account, the Cartesian coordinates of each supercell (***r***) are guaranteed to exist on a 3(*N* – 1) dimensional hyper-plane specified by the fixed center of mass. In other words, eq 7 always creates three singular DOFs in ***r***. These three redundant dimensions were reliably removed from the flow using a *PCA whitening layer*, initialized on Cartesian coordinates of the oxygen atoms from the entire MD data set (after applying eq 7). The whitening transformation was adopted following the approach of ref.^34^ The log volume changes corresponding to the whitening and *un*whitening transformations are  and , respectively, where *n*_mol_ is the number of molecules, and λ_*j*_ > 0 are the eigenvalues of the covariance matrix initialized on the entire MD data set, as detailed in the SI.^34^

### D Reweighting the Learned Distribution and Computing Lattice Free Energies

Using the relevant log-base distributions (eqs 1 and 5, in models C and H respectively), the normalized log-probability ln(*q*(***r***)) of each PGM model (*q*) was defined in the following way:^39^
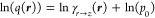
8

In eq 8, ln γ_*r*→*z*_(***r***) represents the log volume change associated with transforming ***r*** to ***z***, as illustrated in Figure 1.^39^

Defining the generalized work function ϕ = *βU* + ln(*q*), one can use the two-state BAR equation (extensively discussed in ref^39^) to estimate *absolute* FE of each macrostate corresponding to a polymorph. Here, one of the two data sets fed into the BAR algorithm is sampled from the PGM mode. At the same time, the other originates from the MD data that samples the equilibrium distribution of a given macrostate. Ergodic samples from the model distribution *q* were obtained by sampling the (uniform) base distribution *p*_0_ and then transforming them via the learned map. Ensemble averages over MD data (⟨•⟩_*p*_) were instead evaluated on locally ergodic configurations, exclusively extracted from the validation data set (i.e., data not included in the PGM training set).^39^ As such, we refer to this estimator as *BAR_V* and indicate the absolute free energy estimates in reduced units obtained from this estimator as , where the subscript *crys* indicates the polymorph.

In each system,  was evaluated on random batches of 10^4^ validation configurations during model training, with a stride of 25 training batches between evaluations. This reweighing approach is both computationally cheap and accurate, an observation that aligns well with results in prior literature.^35,36,39^ Furthermore, the use of the *pymbar* library to evaluate the BAR free energy estimator provides analytical error bars which quantify the uncertainty of  estimates.^51^

### E Averaging Free Energies from Multiple Maps during Training

When training each PGM by maximum likelihood, BAR_V was evaluated multiple times during training, resulting in a set of *M* different *raw* estimates of crystal free energy , that are stochastically distributed around an average, most representative, estimate.

Here, we note that the most accurate of these estimates, i.e., the one associated with lower variance during training by maximum likelihood, coincides with the minimum validation error.^36^ Employing the same notation of ref,^39^ we can label such error when evaluated on the same validation batches as the free energy estimate .^34^

We also note that  increases at the start of training toward a maximum and then decreases gradually with a rate that correlates with overfitting, which our previous work has shown to coincide with increasing variance in the  estimates.^39^

As such, inspired by ref^38^ we adopt the following unsupervised way of averaging multiple  estimates obtained from multiple maps during training:
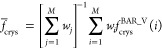
9where weights are proportional to . This approach enables to obtain running weighted averages  that are minimally affected by the large variances seen at the start of training or, in later stages, due to overfitting. Evaluating eq 9 during training yields a converging curve of final FE values (see Figures 3, 4) that in practice takes into account estimates from all the previous versions of the trained maps, and all of the random validation batches encountered. This approach practically extends the *multimap* method described in ref^38^ by introducing a weighting that accounts for model overfitting. In Figures 3 and 4, the standard error bars (*f*^BAR_V^(*i*) ± SE(*i*)) were also averaged using eq 9.

### F PGM Training

**Figure 2:**
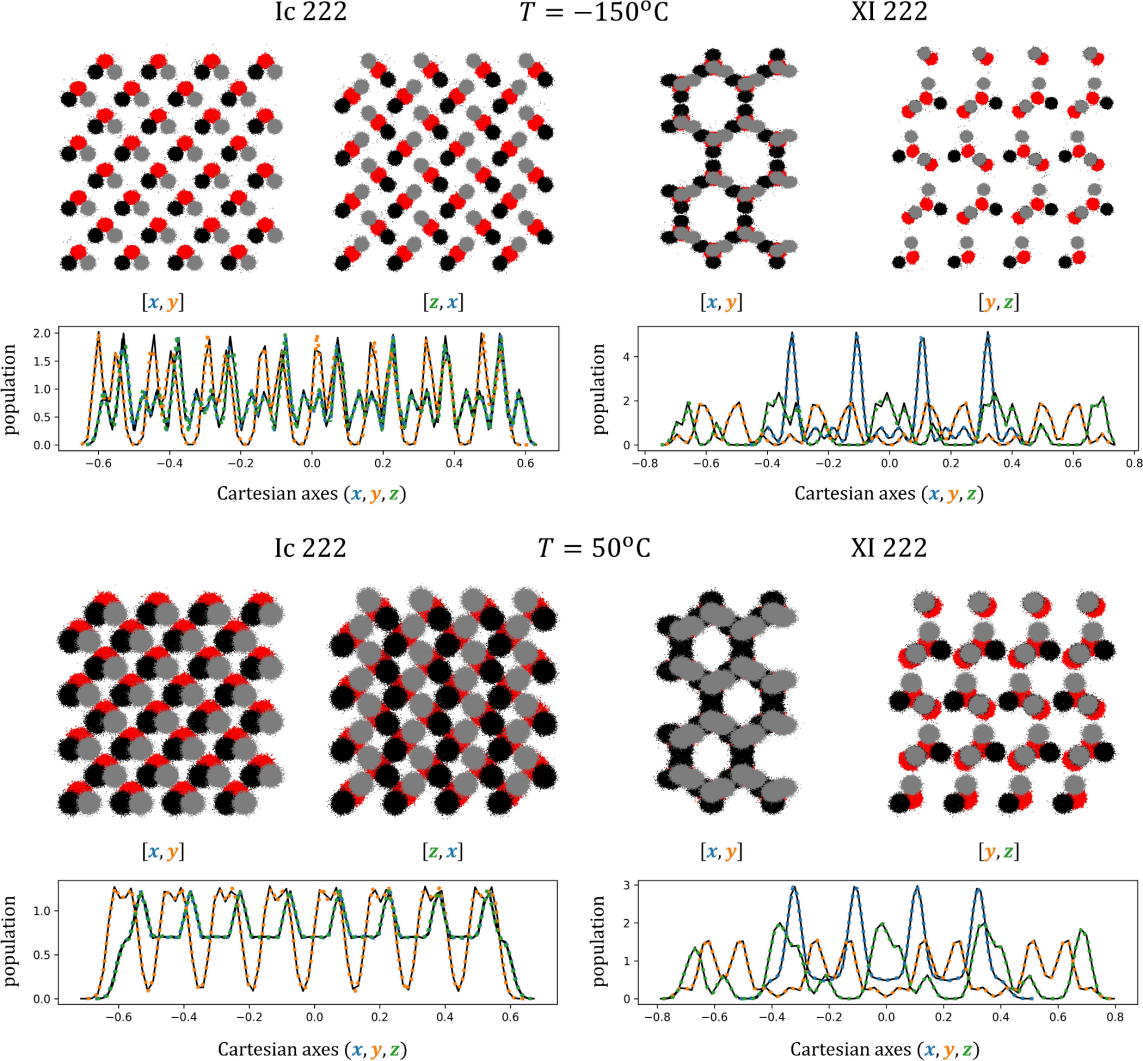
Samples of supercell configurations generated by transforming 20,000 random samples obtained from a uniform *p*_0_, and using four separate instances of model **H**, trained to map configurations of polymorphs XI and Ic at low and high temperature, respectively. The figures show that the four models produce physically consistent samples in the Cartesian space of supercell coordinates. The chosen Ice polymorphs do not exhibit proton disorder. By coloring the hydrogen positions in black and gray, we show that the model can consistently map their coordinates, thus enabling a consistent comparison with the ground-truth method. The probability density plots underneath the system’s configurations report the marginal density obtained from MD data in black solid, and in color, dashed, the marginal probability computed from the mapped configurations, generated transforming *p*_0_. The histograms are virtually indistinguishable, with lines almost perfectly overlapped. An analogous figure showing samples from model **C** is reported in the SI.

Both types of models (**C** and **H**) (see Figure 2) were trained and evaluated in the same way as in our previous work (ref^39^). Specifically, to train a model, the following loss function (eq 11 defined in ref^39^) was minimized via stochastic gradient descent (using Adam optimizer), with respect to the set of model parameters (Θ; located inside the coupling layers; blue in Figure 1):
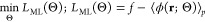
10

The averaging in eq 10 is performed over random batches of the locally ergodic MD training data that are distributed according to the true Boltzmann distribution *p*(***r***) = *Z*^–1^ exp(−*u*(***r***)). The constant of interest *f* = – ln *Z*, appearing eq 10, is unknown prior to performing a FE calculation but does not affect the gradient that is used for training the model ∂_Θ_*L*_ML_. The quantity averaged in eq 10 corresponds to the generalized work function, discussed in section IID. Taking into account eq 8, makes .

### G MD Simulations

Unit cells of most forms of ice can be found in databases such as *American Mineralogist Crystal Structure Database* and *crystallography open database*, in the form of *.cif* files. The specific structures used in this paper are Ic^52^ and XI.^53^ The *cif.* files were converted to *.pdb* files using *Mercury*.^54^ Both unit cells contain 8 molecules. In this paper, we are focused on three system sizes (supercells containing 16, 32, and 64 molecules). The supercells containing these numbers of molecules were obtained by replicating the unit cells by translational stacking. Specifically, if the unit cell is 111, then the two respective sets of supercells of Ic and XI were 211,221,222 and 211,212,222. In creating supercells, we generally prefer supercells that are as close to cubic as possible.

All MD simulations were run using OpenMM^55^ using its TIP4P/Ice force field implementation. The long-range electrostatics were treated using the Particle-Mesh Ewald (PME) algorithm, with an error tolerance parameter of 1e-5, a real space cutoff distance of 0.31 nm, and a switching distance of 0.279 nm. The cutoff (0.31 nm) was chosen considering the shortest box length in supercells 211 and 221 of ice Ic, which was 0.6358 nm.

To sample the NVT ensembles, the *LangevinMiddleIntegrator* integrator, with a friction coefficient of 20 ps^–1^, was used. We note that, at 50 °C, both ice polymorphs are metastable with respect to liquid water; however, within the sampling performed to gather data for training PGM models, no melting events were observed. All simulations were performed with a time step of 2 fs, saving a configuration every 50 timesteps.

Before running the NVT simulations, each of the six supercells mentioned earlier was equilibrated for 1 ns using *MonteCarloAnisotropicBarostat* set to 1 atm at the relevant temperature. Although Open MM does not provide estimates of instantaneous pressure, the average box shapes equilibrated qualitatively well, according to the histograms of the box lengths during the simulation. Out of the 10,000 structures sampled during these simulations, a single structure with a box most similar to the average box was selected as the initial structure for all subsequent NVT simulations.

### H Ground Truth Free Energies with the Einstein Crystal Method

To assess the accuracy of FE estimates obtained from reweighted PGMs, we employed the Einstein Crystal method (ECM) based on ref,^43^ which is representative of the state-of-the-art in estimating fully anharmonic FEs from MD data. In our Open MM-based implementation of the ECM, we adopted a scalar spring constant (*k*), leading to the following potential energy expression:^43^
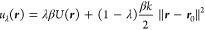
11

In eq 11, λ is a coupling parameter controlling the linear interpolation between the physical ensemble, characterized by potential energy function (*U*), and the ensemble of the Einstein crystal, described by harmonic potential energy function , where ***r***_0_ can be any static configuration of the system with center of mass shifted to zero, and *k* is the scalar spring constant. We set ***r***_0_ to the initial configuration, with the center of mass removed. The spring constant was fixed to 6 × 10^3^ kJ(mol)^−1^(nm)^−2^ so that the Gaussian distributions associated with the harmonic potential have negligible periodic images along the smallest box length (0.6358 nm).^43^ The ground truth FE of a given supercell (*f*_crys_^ECM^) is then estimated as^43^
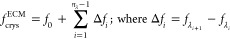
12where  are normalized masses *m*_*i*_ of the atoms in the system. In this work, *n*_λ_ = 20 separate simulations were ran with λ parameters λ_1_, ..., λ_20_ taking on values 0, ..., 1, respectively. The intermediate values, arranged in ascending order between zero and one, are reported in SI Figure 6(A). The same set of lambda values were used in all systems. Since the λ_1_ = 1 simulation samples the translationally invariant ensemble of the unperturbed system of interest, all other simulations were initialized from ***r***_0_ with *CMMotionRemover* functionality of *openMM* being active. This removes the average momentum of the center of mass during the simulations. Additionally, every 50 timesteps during these simulations, the center of mass was actively removed from the positions (***r***) to make sure that all systems remained on the same 3(*N* – 1) dimensional subspace. In eq 12, Δ*f*_*i*_ was calculated *n*_λ_ – 1 = 19 times using BAR functionality of *pymbar*.^51^ This was done several times during the simulations to show how the FE estimates converge as a function of more data becoming available for the BAR calculations (SI Figure 6(B)). The set of *ground truth* FEs reported in this paper utilized all of the available data. The standard error of *f*_crys_^ECM^ was estimated by summing the *n*_λ_ – 1 intermediate standard errors provided by *pymbar*. This approach provides a lower-bound estimate of the uncertainty on *f*_crys_^ECM^, because this error estimate assumes complete decorrelation between the samples used to compute free energy differences between neighboring replicas. While underestimating the uncertainty of the ground-truth method, this approach is fast (i.e., does not require bootstrapping) and provides a relatively more ambitious target with which to compare the PGM-based methods. Finally, *f*_0_ appearing in eq 12 is defined as
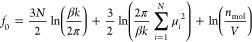
13and takes three additive terms, including the FE of the Einstein crystal, the FE associated with the removal of the center of mass from the Einstein crystal, and the FE associated with removing the center of mass from the unperturbed physical system (where *V* was set to the volume of the box).^43^ Please refer to ref^43^ for further details about these terms.

Using the ground truth method described above (ECM), a total of 1.84 μs of data was sampled, with 0.32 μs of this data (17.4%) sampling the physical ensembles (i.e., λ = 1). This percentage is >5% because each physical simulation was four times longer than any other simulation of the same ECM run to collect sufficient MD data for later use in the PGM models. Collecting this data in the context of ECM ensured that the average potential energy in each system was the same across all FE methods being compared in this work. Notably, one major methodological discrepancy between the ground truth method and the PGMs can be linked to how the center of mass was removed. In the EC calculations, the center of mass of the supercells was removed based on the position of all atoms (including the hydrogens). Meanwhile, in the PGMs, the center of mass being removed was only based on the oxygen atoms, a more practical choice given the fact that the oxygens are used to anchor Cartesian blocks (eq 7). This methodological difference does introduce small systematic discrepancies between the *absolute* free energy of supercells that cancel out when considering free energy differences between polymorphs, as shown in the Results section.

## III Results

In this section, we quantitatively assess the results from the two types of flow-based PGMs, i.e., models **C** and **H**, introduced in sections IIA and IIB, respectively. The ground truth estimates of the lattice free energy were obtained using the Einstein crystal method (ECM), introduced in section IIH.

**Figure 3:**
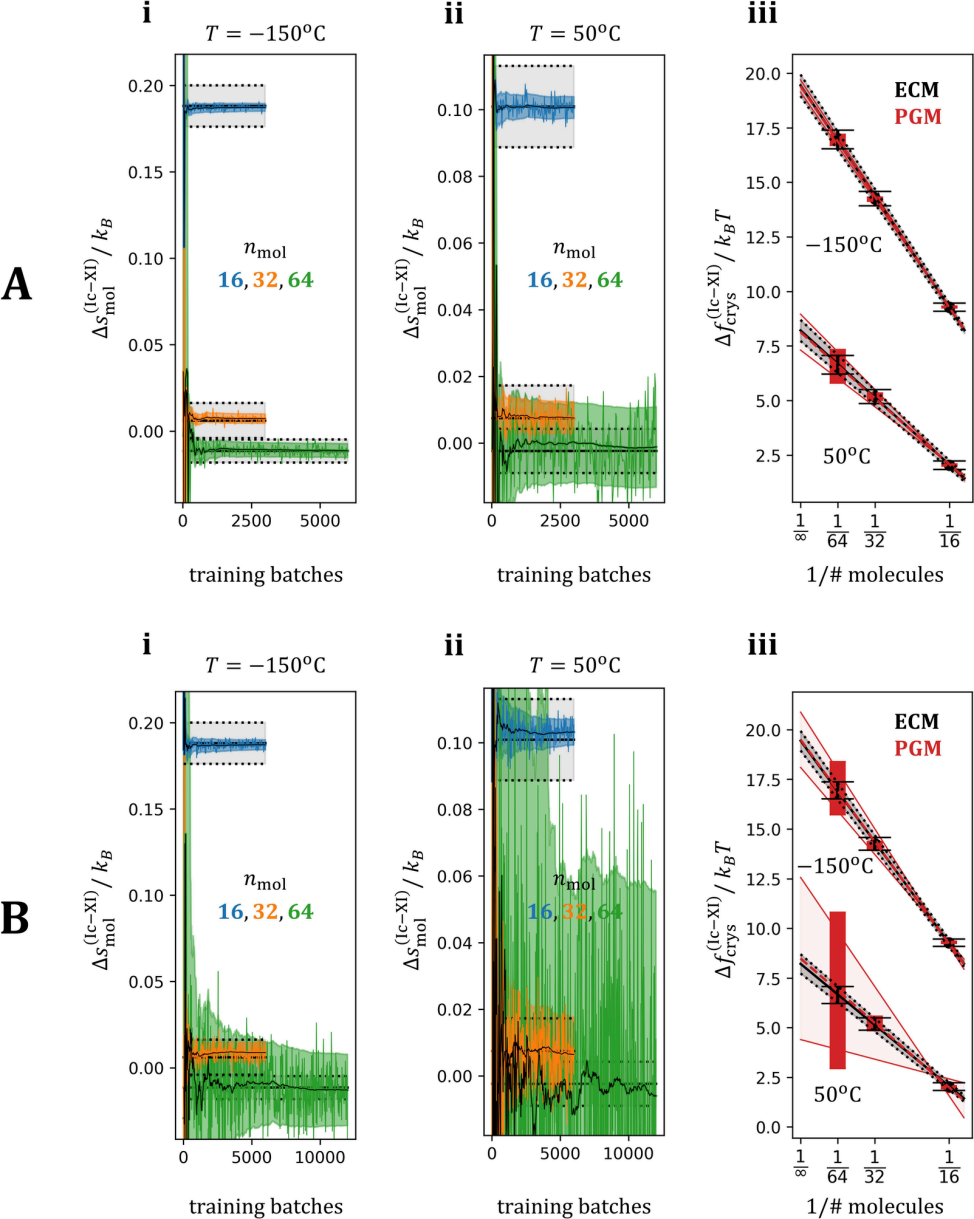
Free energy and entropy differences between ice polymorphs. A: model **H**, B: model **C**. The straight, black, solid colored lines represent reference ground truth computed with the ECM. The corresponding error bars are shown using straight black dotted lines. For cases where the running estimate of the PGM-based method renders it difficult to assess the ground truth accurately, we refer the reader to Table 1. Plots labeled with *i* and *ii* report the entropy differences per molecule between forms Ic and XI as a function of the number of training batches (i.e., during training). The estimates from models **H** and **C** are shown using colors corresponding to the three system sizes investigated in this study. The fluctuating colored lines indicate the *raw* estimates from the models, and the fluctuating, converging black curves indicate the cumulative weighted averages of these *raw* estimates, obtained as discussed in section IIE). Plots labeled *iii* display the differences in crystal-free energy between the two forms, with a line of best-fit extrapolating to approximate the same quantities in the thermodynamic limit (y-intercept). All transparent areas represent standard error bars (colored: PGM models, black: ECM ground truth).

**Figure 4:**
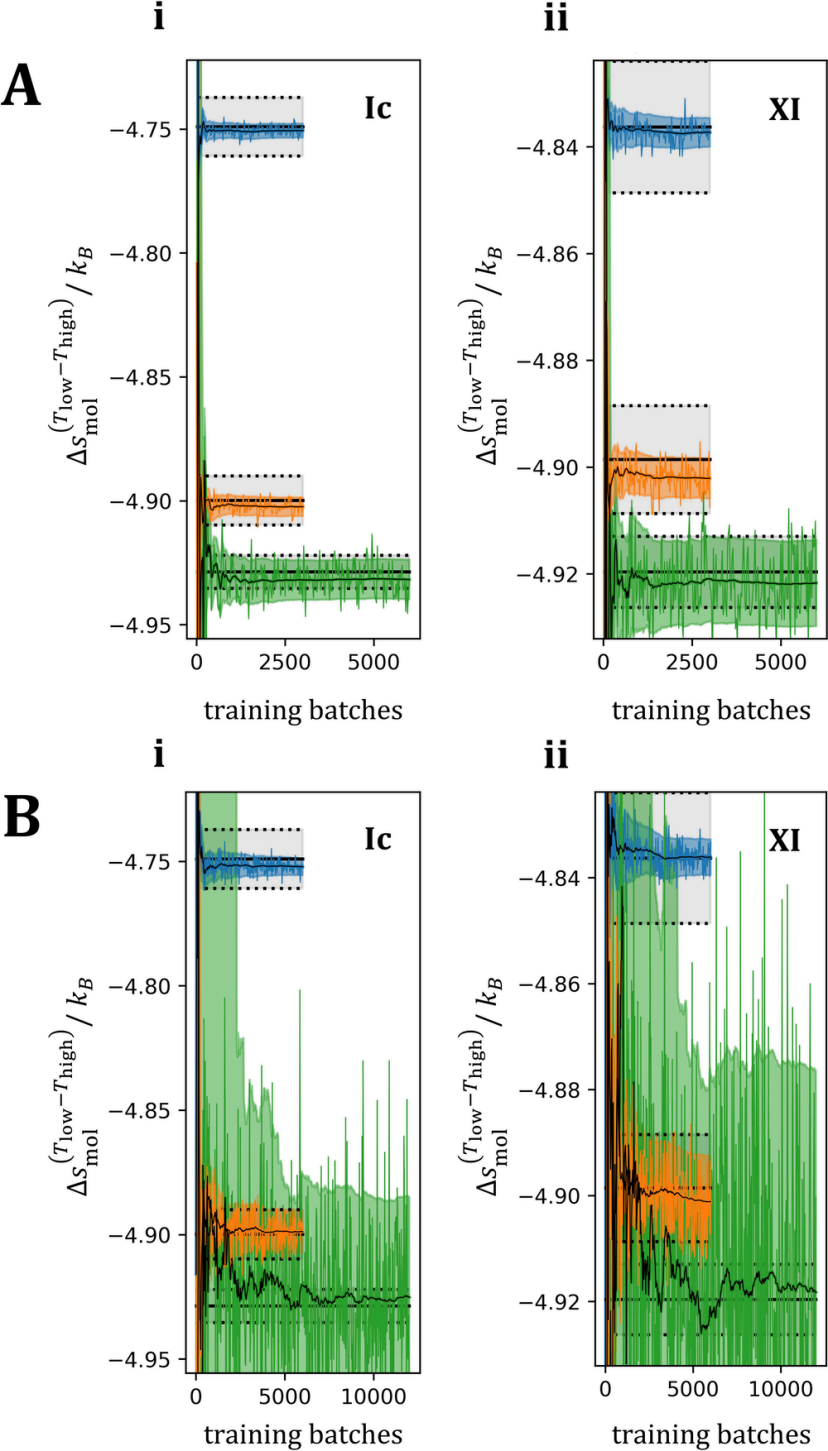
Free energy differences across temperatures, rather than across polymorphs as reported in Figure 3. A: model **H**, B: model **C**. The solid, straight black lines represent the ground truth estimates (from ECM). Plots labeled with *i* and *ii* report the entropy differences per molecule between two versions of the same systems at the two temperatures (*T*_low_ = −150 °C and *T*_high_ = 50 °C), as a function training progress. The estimates from models **H** and **C** are represented using colors corresponding to the three system sizes investigated in this study. The fluctuating colored curves are the *raw* BAR_V estimates on random subsets of 10,000 points from the validation set, and the black curves are the cumulative weighted averages of these *raw* estimates, obtained as discussed in section IIE). All transparent regions represent standard error bars (colored: PGM models, black: ECM ground truth).

Two types of plots are used to visualize the comparisons between methods. Plots showing temperature-reduced crystal free energy differences Δ*f*_crys_^(A - B)^ = *f*_crys_^(A)^ – *f*_crys_^(B)^, and plots showing temperature-reduced entropy differences per molecule Δ*s*_mol_^(A - B)^ = *s*_mol_^(A)^ – *s*_mol_^(B)^, where (A, B) are either the two different Forms of ice (Ic, XI) (Figure 3) or the two temperatures (−150 °C, 50 °C) (Figure 4).

The average potential energy in each state ⟨*U*⟩_*i*_ is constant across all methods because it is obtained from the same underlying set of MD-generated configurations. Hence, by comparing the entropic contribution to the lattice free energy *s*_*i*_, we introduce a more stringent criterion to assess the accuracy of free energy estimates from PGMs trained by likelihood, akin to our previous work.^39^

The per-molecule temperature-reduced entropy of polymorph *i* is defined as
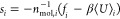
14where state *i* is specified by the identity of the metastable state (Form) and the physical conditions of the given Canonical ensemble (*N*, *V*, *T*).

Table 1, based on raw data reported in SI Figure 5, reports the lattice Helmholtz free energy differences (in units of *k*_*B*_*T*) between the two forms of ice, computed using all of the methods (i.e., from models **H** and **C**, and from the ECM). These comparisons show a significant level of agreement between the different methods, indicating that the results from the PGMs are accurate. We note, however, that the error bars are higher in larger systems and at higher temperatures because such ensembles occupy a larger volume of configurational space. That being said, we consistently observe smaller error bars from results of model **H**, compared to model **C**. This indicates that model **H** (based on the decoupled, quaternion-based representation of the Cartesian blocks) is better suited for efficiently learning configurational distributions of ice crystals. Moreover, since both models we compare used the same architecture for the trainable part of the model (spline coupling layers; see Figure 1), we expect that the computational gains from using the decoupled representation of the Cartesian block can be generalized to other types of crystals.

**Table 1:** Final Helmholtz Free Energy Differences (in units of *k*_*B*_*T*) with Their Associated Standard Errors, between Ice Ic and XI Obtained Using Two PGM Models, **H** and **C**, and the ECM^a^

Δ_*Ic*–*XI*_ @ *T* = −150 °C					
*n*_*mol*_	Δ⟨*f*⟩_*ECM*_	Δ⟨*f*⟩_**H**_	|Δ⟨*f*⟩_*ECM*_ – Δ⟨*f*⟩_**H**_|	Δ⟨*f*⟩_**C**_	|Δ⟨*f*⟩_*ECM*_ – Δ⟨*f*⟩_**C**_|
16	9.27 ± 0.19	9.29 ± 0.04	0.014 ± 0.23	9.29 ± 0.05	0.016 ± 0.24
32	14.25 ± 0.33	14.22 ± 0.09	0.033 ± 0.41	14.17 ± 0.18	0.084 ± 0.51
64	16.95 ± 0.43	16.94 ± 0.26	0.009 ± 0.68	17.05 ± 1.32	0.099 ± 1.75
*∞*	19.44 ± 0.49	19.41 ± 0.28	0.033 ± 0.78	19.49 ± 1.39	0.048 ± 1.89

Δ_*Ic*–*XI*_ @ *T* = 50 °C					
*n*_*mol*_	Δ⟨*f*⟩_*ECM*_	Δ⟨*f*⟩_**H**_	|Δ⟨*f*⟩_*ECM*_ – Δ⟨*f*⟩_**H**_|	Δ⟨*f*⟩_**C**_	|Δ⟨*f*⟩_*ECM*_ – Δ⟨*f*⟩_**C**_|
16	2.03 ± 0.20	2.04 ± 0.05	0.005 ± 0.25	2.00 ± 0.06	0.037 ± 0.26
32	5.18 ± 0.32	5.18 ± 0.15	0.002 ± 0.47	5.21 ± 0.36	0.033 ± 0.67
64	6.64 ± 0.42	6.56 ± 0.77	0.075 ± 1.20	6.87 ± 3.93	0.234 ± 4.36
*∞*	8.21 ± 0.48	8.13 ± 0.82	0.078 ± 1.31	8.48 ± 4.08	0.269 ± 4.57

a The accuracy of the PGM estimates is assessed by computing their absolute deviation with respect to the ECM ground truth as |Δ⟨*f*⟩_*ECM*_ – Δ⟨*f*⟩_*i*_|, where *i* = **H**, **C**. This analysis indicates that while agreement is excellent across all models, model **H** estimates are closer to ECM despite being trained on the same data. The underlying values of *absolute* FE estimates for ice Ic and XI are reported in SI Figure 6.

Figure 3 reports per-molecule, temperature-reduced entropy difference (in units of *k*_*B*_) between the two forms of ice (Ic and XI), estimated using the different methods. In these comparisons, the two temperatures are plotted separately. Figures 3A (i, (ii) and Figure 3B (i,ii) report estimates obtained from the PGM models **H** and **C** respectively, plotted as a function of the training progress. The differences between panels A and B in Figure 3 indicate that model **H** was able to converge more rapidly and with lower variance compared to model **C**. These estimates are systematically comparable to the ECM ground truth (straight black lines).

The reason for the lower variance in the BAR_V estimates from model **H**, compared to model **C**, can be linked to the differences in the amount of overlap observed between the potential energies of the data and the potential energies of the samples drawn from the models during training (shown in SI Figures 2 and 3). When taken together, the current results show that in the large systems containing 64 molecules and at the higher temperature (50 °C) (green error bars in Figure 3A and B, (ii), both models tend to approach a bottleneck in accuracy.

Both models **H** and **C** enable us to consistently extrapolate the lattice free energy to the thermodynamic limit, achieving results consistent with the ECM ground truth (see Figure 3A and B, (iii). Also, in this case, the lower error associated with model **H** is evident, and the level of accuracy in the high-temperature simulations is of the same order as the ECM ground truth.

Figure 4 reports per-molecule, temperature-reduced entropy differences between low and high-temperature ensembles of the same systems, based on the estimates from the three methods (PGM models: **H** (panel A) and **C** (panel B), and the ground truth method: ECM (all panels)). These estimates are plotted as a function of training progress. Also, in this case, the agreements between the PGM-based and the ground truth (ECM-based) estimates of the temperature-dependent entropy difference in the three system sizes are significant. As for the comparison between polymorphs, estimates obtained from model **H** are associated with significantly smaller error bars than the estimates from model **C**, once again underscoring the importance of adopting a physically motivated representation in constructing flow-based maps for targeted FE calculations.

For completeness, samples drawn from the larger models (with 64 molecules) are visualized in Figure 2 and SI Figure 4. There are no significant qualitative differences between the two types of models (model **C** vs **H** respectively).

Our results clearly show that both the computational costs associated with training and the numerical precision of the resulting free energy estimates obtained from flow-based PGMs, even when accurately reweighted,^39^ are strongly affected by the representation of the relevant DOFs.

## IV Discussion and Conclusions

In this work, we have demonstrated that given an accurate reweighting scheme,^39^ the lattice free energy differences between packing polymorphs of flexible ice crystals, modeled using a classical force field (TIP4P-ice), can be efficiently computed using *targeted* free energy calculations based on normalizing flow models, remapping from a common, flat, analytical base distribution.

Demonstrating the method with a uniform base distribution is significant because it can be generalized to model permutationally invariant distributions, which has been a highlight in prior literature.^26,27,30,31^ Furthermore, training with a flat base distribution generates flows that can be easily composed back-to-back to connect any pair of ensembles of the same dimensionality, thus opening up the possibility of connecting different packings to a *common* base distribution. This, in turn, enables reweighting with Multistate BAR, as discussed in ref^38^ and demonstrated for isolated molecules in ref.^39^ Furthermore, mapping across packing polymorphs via a flat base distribution enables training a number of maps equal to the number of metastable states, thus scaling favorably with the number of polymorphs investigated and setting the scene for large-scale applications of free energy methods based on PGMs. The results illustrated here show that mapping via a flat base distribution is not only possible but also accurate and computationally cheaper than ground truth with the whole range of temperature conditions of practical relevance for finite-temperature crystal structure prediction.

Our results show that this approach can handle molecular crystals where all the internal degrees of freedom of a given Cartesian block are explicitly modeled. As such, it is a representative example of how Cartesian blocks can be treated in larger molecules. We, therefore, believe that the current work is a useful stepping stone toward assessing the applicability of PGM-based free energy methods to complex and realistic crystal systems, including molecular crystals with multiple conformational degrees of freedom.^6^

Across all tests performed in this work, we note how the extrapolation to the thermodynamic limit of an infinitely large cell shows a convincingly convergent behavior enabling a quantitative removal of finite-size effects from both lattice-free energy differences and entropies. The order of magnitude of the uncertainty is particularly important in these estimates. Our current setup showed that a PGM-based approach such as model **H** enables matching the ground-truth error levels.

We note that the PGM approach is more computationally efficient than the Einstein crystal method (ECM), as it requires performing only two simulations of the physical end-states of interest vs multiple simulations in perturbed Hamiltonians necessary for converging the ECM. The difference in cost can be quantified by comparing the amount of MD-generated samples used by the two methods. In this regard, PGM requires only ≈17% of the configurations needed by the ECM, leading to an increase in MD efficiency of factors between 5 and 6. Given that the FEs from PGMs in the current work have converged quite quickly during training, we anticipate that further efficiency gains can be reached in more complex molecular systems.

We further note that the computational overheads associated with training PGMs by maximum likelihood are *agnostic* to the quality and computational cost of the potential energy functions used to sample the training data. This opens up future possibilities of computing fully anharmonic free energy differences, remapped to force fields at a higher level of theory.^37,38^

The choice of a common base distribution, the application to FE differences in a wide and practically relevant range of temperatures, the flexible representation of individual molecules, and the application to packing polymorphs with nonoverlapping high-temperature distributions provide a step forward with respect to the literature on PGM-based FE calculations on molecular crystals.^30^ As such, these results represent a stepping stone toward adopting normalizing flow methods into the anharmonic FE toolbox of practitioners dealing with finite temperature ranking of large sets of molecular crystalline solids. More broadly, when taken together with several key recent developments in this field,^28,31^ we can further expect normalizing flows to begin solving increasingly diverse real-world problems in the foreseeable future.

## Supporting Information Available

Figure reporting a representative coupling pattern between marginals, Analysis of the numerical error associated with inversion, Description of the whitening approach, potential energy overlaps obtained during training, qualitative comparison of configurations generated by PGM and MD simulation, convergence of the ECM calculations and spacing of the λ replicas, table summary of the absolute FE of every supercell (PDF)
