# Supplementary material for: Accurate Lattice Free Energies of Packing Polymorphs from Probabilistic Generative Models
Source: J Chem Theory Comput. 2025 Feb 21;21(5):2244–55. doi: 10.1021/acs.jctc.4c01612 (PMC11912200; doi:10.1021/acs.jctc.4c01612)
Supplement: Supplementary file 1 — ct4c01612_si_001.pdf [file ct4c01612_si_001.pdf]

Supplementary Materials for: Accurate Lattice Free Energies of Packing Polymorphs from Probabilistic Generative Models

Edgar Olehnovics,[1] Yifei Michelle Liu,[2] Nada Mehio,[3] Ahmad Y. Sheikh,[3] Michael R. Shirts,[4] and Matteo Salvalaglio[1]

[1] *Thomas Young Centre and Department of Chemical Engineering, University College London, London, WC1E 7JE, United Kingdom.*

[2] *Molecular Profiling and Drug Delivery, Research Development, AbbVie Bioresearch Center, Worcester, MA, 01605, USA*

[3] *Molecular Profiling and Drug Delivery, Research Development, AbbVie Inc, North Chicago, IL, 60064, USA*

[4] *University of Colorado Boulder, Boulder, CO, 80309, United States*

(*m.salvalaglio@ucl.ac.uk)

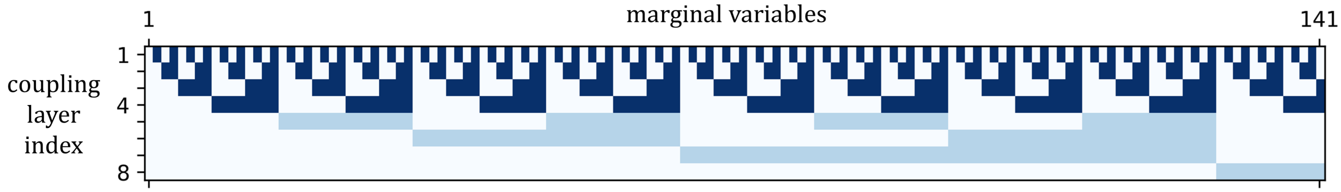

Figure S1: Coupling patterns in the case of crystal containing 16 flexible water molecules (141 degrees of freedom). In each row (coupling layer): dark-coloured marginal variables are used to condition the transformation of light-coloured variables, and then light-coloured variables are used to condition the transformation of dark-coloured variables. To represent correlations between all marginal variables, the full set of coupling layers can be used (8 in this case).[1] In model H, number of coupling layers was fixed to the top 4. Model C used all coupling layers (i.e., 8,9,10 for 16,32,64 water molecules, respectively).

S1. NUMERICAL ERRORS OF INVERTING THE FLOWS

The following SI Figure 8 was created after generating the PGM results reported in the main text, but it serves as a representative illustration of the magnitudes of numerical errors associated with inverting data through either type of flow architectures that were compared in the current work. Importantly, any bijective functions (bijectors) used for targeted free energy perturbation must be invertible on any data points, in both directions, without any error. This must be true in both the coordinates, and in the associated changes of infinitesimal volume γ, to conform to the underlying theory. In SI Figure 8, we can see that these conditions are not strictly met for all BAR_V inputs during the training process. This can be due to a sub-optimal initial choice of bijective functions or due to the sub-optimal implementation of otherwise very accurate bijectors. Both factors could be involved in the current work, however according to SI Figure 8, model **H** on average performed much better than **C** in terms of invertibility, in the current systems. This is likely because in SI Figure 8, model **H** and **C** used 4 and 9 spline coupling layers, respectively, allowing a larger amount of error to accumulate, originating from the chosen hyperparameters and floating point resolution that was used in the 1D splines in the current work. On the other hand, using fewer coupling layers in model **C** was associated with larger error bars on the FE estimates due to a relatively limited ability of the model train on raw Cartesian coordinates under the same set-up hyperparameters compared to model **H**. Further work is needed to minimised the types of errors seen in SI Figure 8, however the core conclusions of the current paper are unaffected by this.

S2. WHITENING

The following equation describes the general form of the whitening layers, where the singular value decomposition (SVD) algorithm can be used to diagonalise the covariance matrix C_{ij}.[2] This covariance matrix, and the average supercell $\langle r_i \rangle$, must be initialised a-priori on all of the available MD data that is later intended for training and evaluating the model.

$$
\begin{aligned}
x_j &= \sum_{i=1}^{D} (r_i - \langle r_i \rangle) U_{ij} \lambda_j^{-1/2} \quad ; \quad \lambda_j > 0 \\
r_i &= \sum_{j=1}^{D-d_{\text{removed}}} U_{ij} \lambda_j^{1/2} x_j + \langle r_i \rangle \\
&\text{where } ; \; U_{ij}\lambda_j U_{kj} = C_{ik} = \langle (r_i - \langle r_i \rangle)(r_k - \langle r_k \rangle) \rangle
\end{aligned}
\tag{S1}
$$

In Eq. S1, the Cartesian coordinates are labeled with $r_i = \boldsymbol{r}$, the whitened coordinates are labeled with $x_j = \boldsymbol{x}$, and eigenvalues of the covariance matrix are labeled using λ_j. The number of marginal variables in $\boldsymbol{x}$, compared to $\boldsymbol{r}$, is reduced by d_{removed}, where d_{removed} is equal to the number of eigenvalues that are too small (singular). Since oxygen centred of mass was removed earlier, it was observed that $d_{\text{removed}} = 3$ was consistently the only valid option, provided that the Cartesian coordinates chosen to be whitened include all of the oxygen atoms. In the current work, Eq. S1 was used to transform only between the physical Cartesian coordinates of the oxygen atoms ($\boldsymbol{r}_{\text{O}} \in \mathbb{R}^{3n_{\text{mol}}}$) and the whitened *Cartesian* coordinates of oxygen atoms $\boldsymbol{c}_{\text{O}} \in \mathbb{R}^{3(n_{\text{mol}}-1)}$, as labeled in both models in Figure 1 in

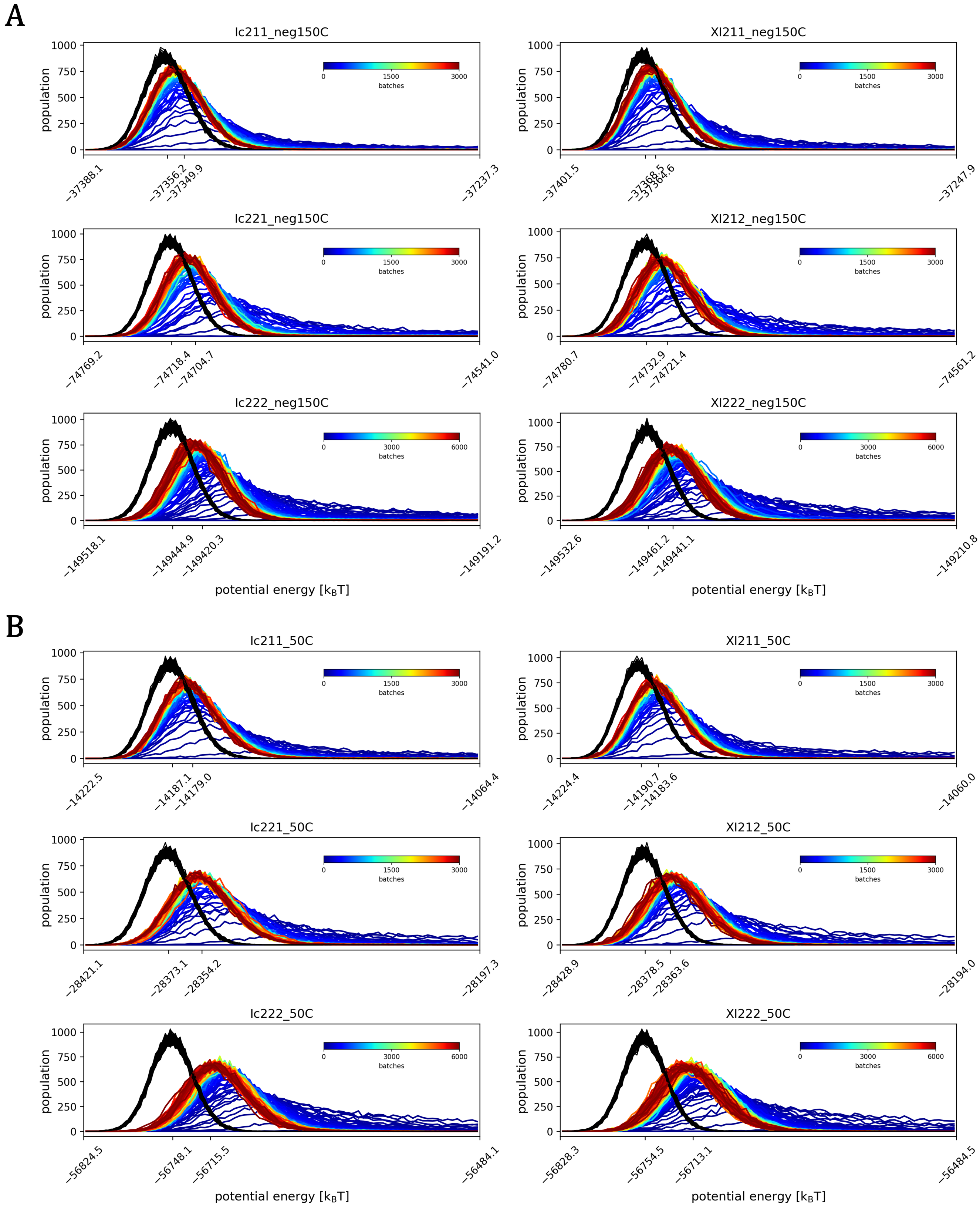

Figure S2: Histograms of potential energies of samples drawn from model **H** during training. With a stride of 25 training batches, 10,000 conformers underlie each histogram. Coloured histograms represent (unweighted) populations of potential energies of supercells sampled from different instances of model H during training on the different systems. (A) high temperature systems (50°C). (B) low temperature systems (-150°C). Each black histogram corresponds to 10,000 random conformers in the corresponding training and validation batches. This figure is based on the same data that was used for FE calculations (using BAR_V), reported in the main text.

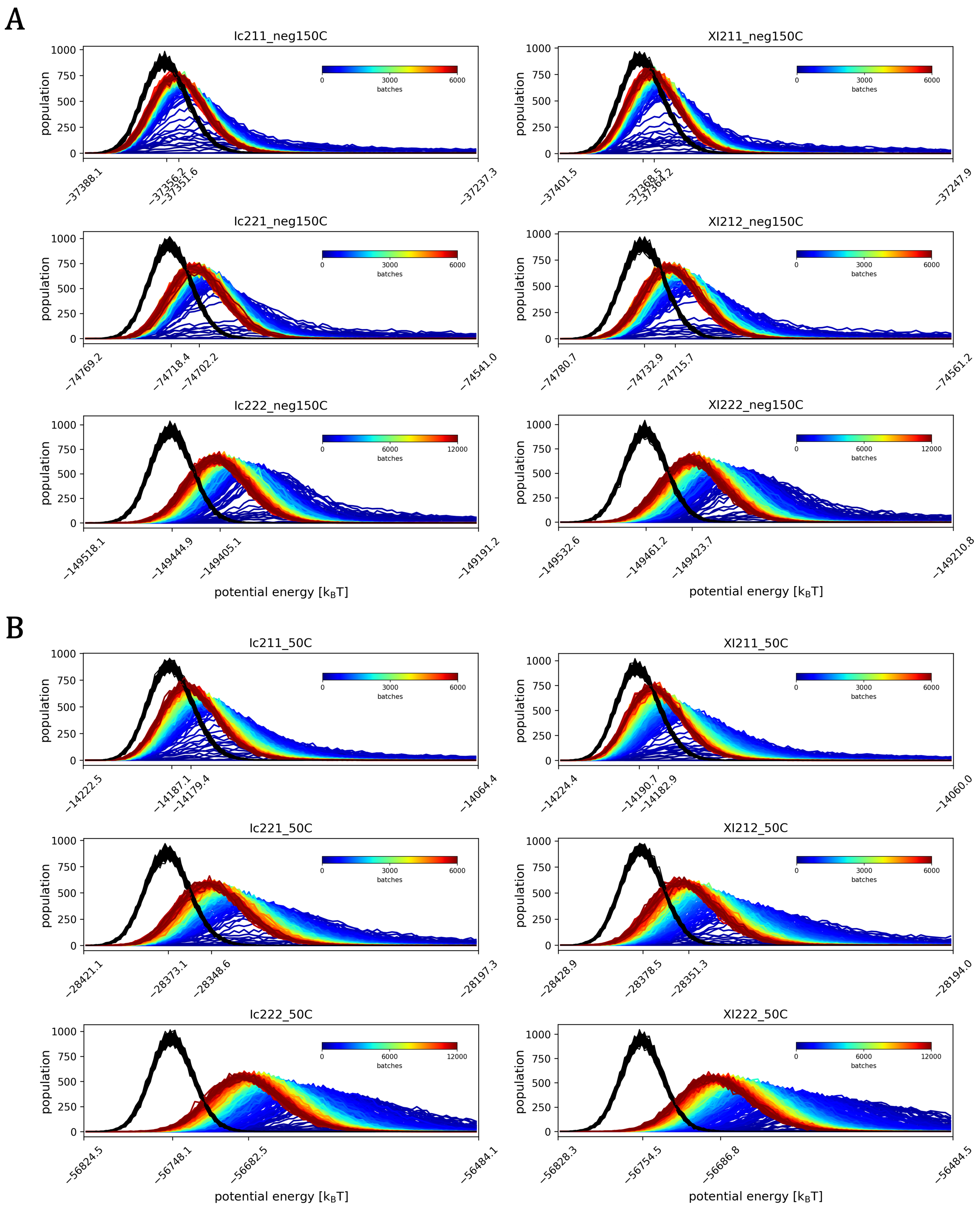

Figure S3: Histograms of potential energies of samples drawn from model **C** during training. With a stride of 25 training batches, 10,000 conformers underlie each histogram. Coloured histograms represent (unweighted) populations of potential energies of supercells sampled from different instances of model H during training on the different systems. (A) high temperature systems (50°C). (B) low temperature systems (-150°C). Each black histogram corresponds to 10,000 random conformers in the corresponding training and validation batches. This figure is based on the same data as used for FE calculations (using BAR_V), reported in the main text.

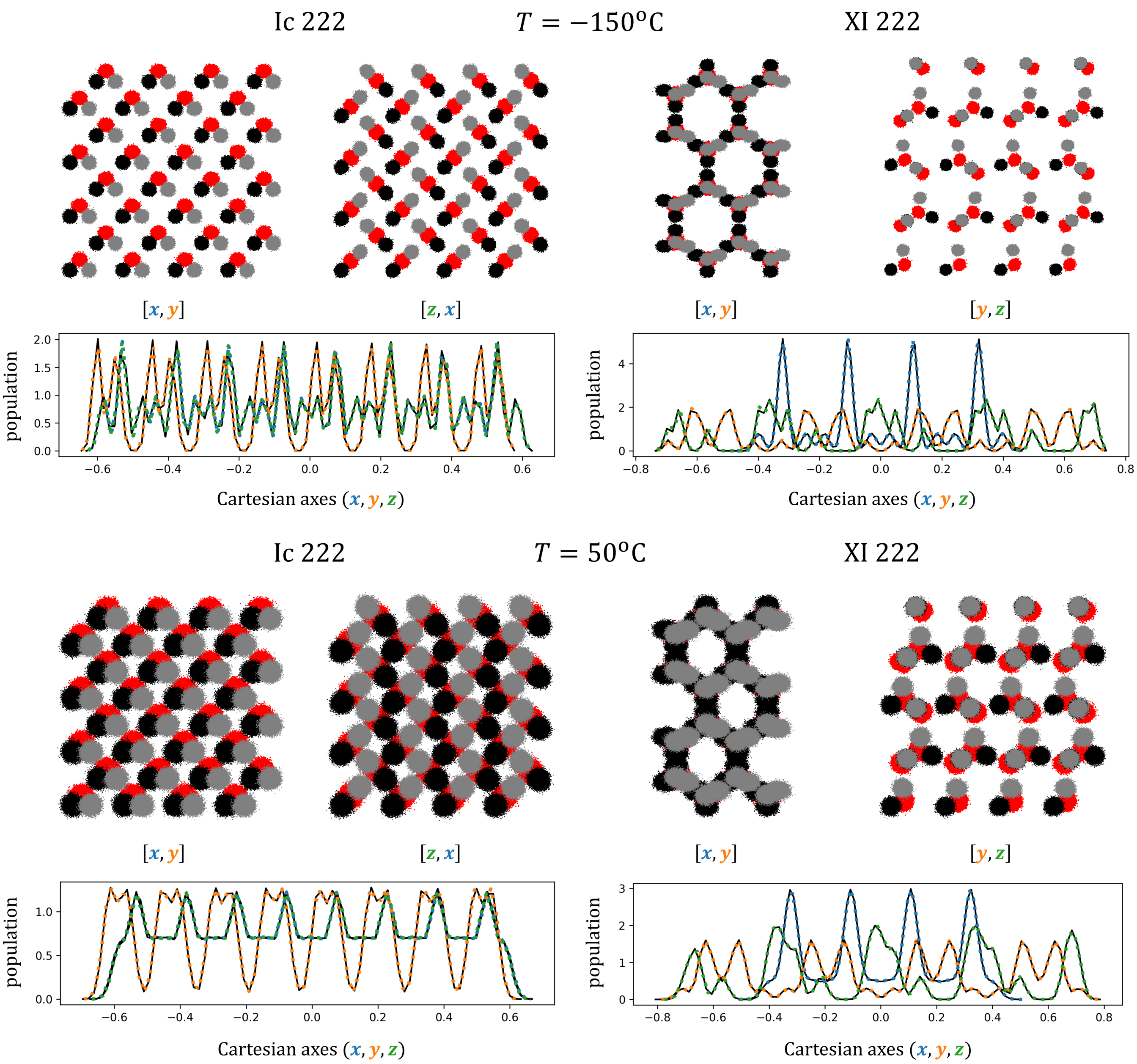

Figure S4: Samples of supercell configurations generated by transforming 20,000 random samples from a uniform base distribution (p_0) using four separate instances of model **C**, after training. The figures correspond to four of the systems investigated, showing that qualitatively, the four models produced plausible samples in the Cartesian space of supercell coordinates (scatter plots and the dashed coloured histograms), matching the configurational distributions in the training data (black histograms). A comparison with an analogous figure for model **H** shows that a visual appearance of the samples is useful to check but does not guarantee better quantitative accuracy. .

the main text. Since the matrix U_{ij} in Eq. S1 is orthogonal ($\det(U_{ij}) = 1$), the log volume changes corresponding to the whitening and unwhitening transformations are $-\frac{1}{2}\sum_{j=1}^{D-3}\ln\lambda_j$ and $\frac{1}{2}\sum_{j=1}^{D-3}\ln\lambda_j$, respectively.[3]

---

[1] Christina Gao, Joshua Isaacson, and Claudius Krause. i-flow: High-dimensional Integration and Sampling with Normalizing Flows. *Machine Learning: Science and Technology*, 1(4):045023, November 2020. arXiv:2001.05486 [hep-ph, physics:physics,

Ic		T = -150C						
cell	n_{mol}	$\langle u \rangle$	ECM		H		C	
211	16	-37356.2242	-36785.2478	0.0934	-36785.2694	0.0187	-36784.6620	0.0233
221	32	-74718.4300	-73565.4764	0.1603	-73565.1498	0.0450	-73563.9848	0.0913
222	64	-149444.9315	-147130.9626	0.2135	-147130.9328	0.1299	-147128.3638	0.6395
**XI**								
cell	n_{mol}							
211	16	-37368.5064	-36794.5191	0.0977	-36794.5544	0.0172	-36793.9494	0.0230
212	32	-74732.8801	-73579.7290	0.1647	-73579.3691	0.0424	-73578.1531	0.0910
222	64	-149461.1517	-147147.9127	0.2135	-147147.8736	0.1266	-147145.4124	0.6848

Ic		T = 50C						
cell	n_{mol}	$\langle u \rangle$	ECM		H		C	
211	16	-14187.0635	-13692.0716	0.0955	-13692.1183	0.0261	-13691.5358	0.0310
221	32	-28373.1182	-27376.9605	0.1565	-27376.7158	0.0758	-27375.4341	0.1743
222	64	-56748.0501	-54749.5163	0.2122	-54749.6871	0.3828	-54746.6949	1.9401
**XI**								
cell	n_{mol}							
211	16	-14190.7131	-13694.1065	0.0998	-13694.1578	0.0259	-13693.5339	0.0305
212	32	-28378.5354	-27382.1384	0.1586	-27381.8920	0.0783	-27380.6453	0.1853
222	64	-56754.5379	-54756.1541	0.2119	-54756.2498	0.3897	-54753.5667	1.9923

Figure S5: Table of *absolute* Helmholtz free energies (in units of $k_B T$) provided by the three methods (the two PGM models; **H** and **C**, and the ground truth method; ECM). The standard errors of each estimate are shown to the right of the estimate.

stat].
[2] noegroup/bgflow, January 2024. original-date: 2021-04-15T15:37:40Z.
[3] Frank Noé, Simon Olsson, Jonas Köhler, and Hao Wu. Boltzmann generators: Sampling equilibrium states of many-body systems with deep learning. *Science*, 365(6457):eaaw1147, September 2019. Publisher: American Association for the Advancement of Science.

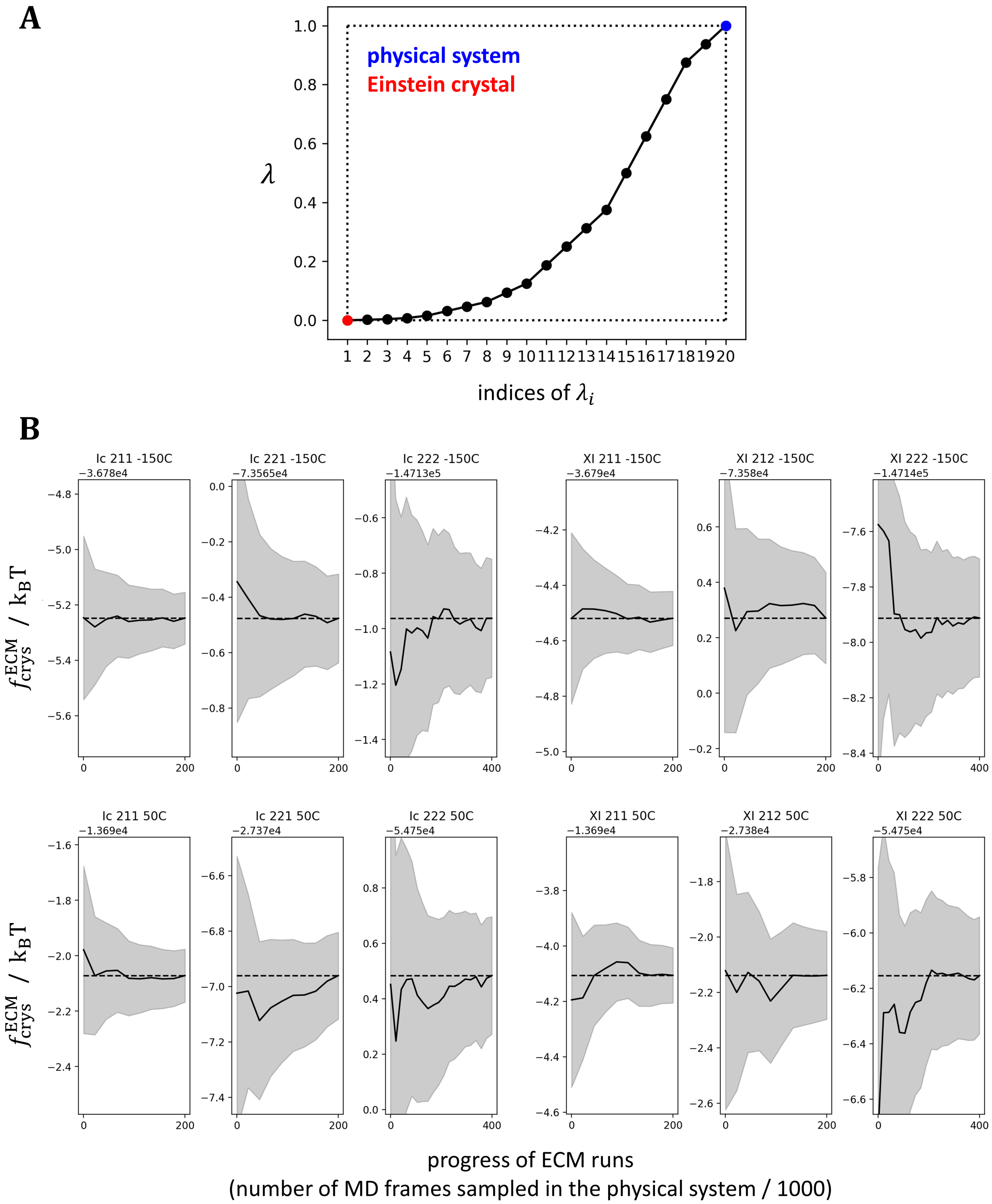

Figure S6: ECM runs. (A) Exact 20 lambda values used in all systems were: [0., 0.00195, 0.003905, 0.0078125, 0.015625, 0.03125, 0.046875, 0.0625, 0.09375, 0.125, 0.1875, 0.25, 0.3125, 0.375, 0.5, 0.625, 0.75, 0.875, 0.9375, 1.] (B) The final FE value (dashed horizontal line) was taken as ground truth. Standard error bars are shown, decreasing with more data as anticipated. In each run, the physical (unperturbed) system sampled 4 times more data than the 19 perturbed systems (including the state of the Einstein crystal with removed centre of mass).

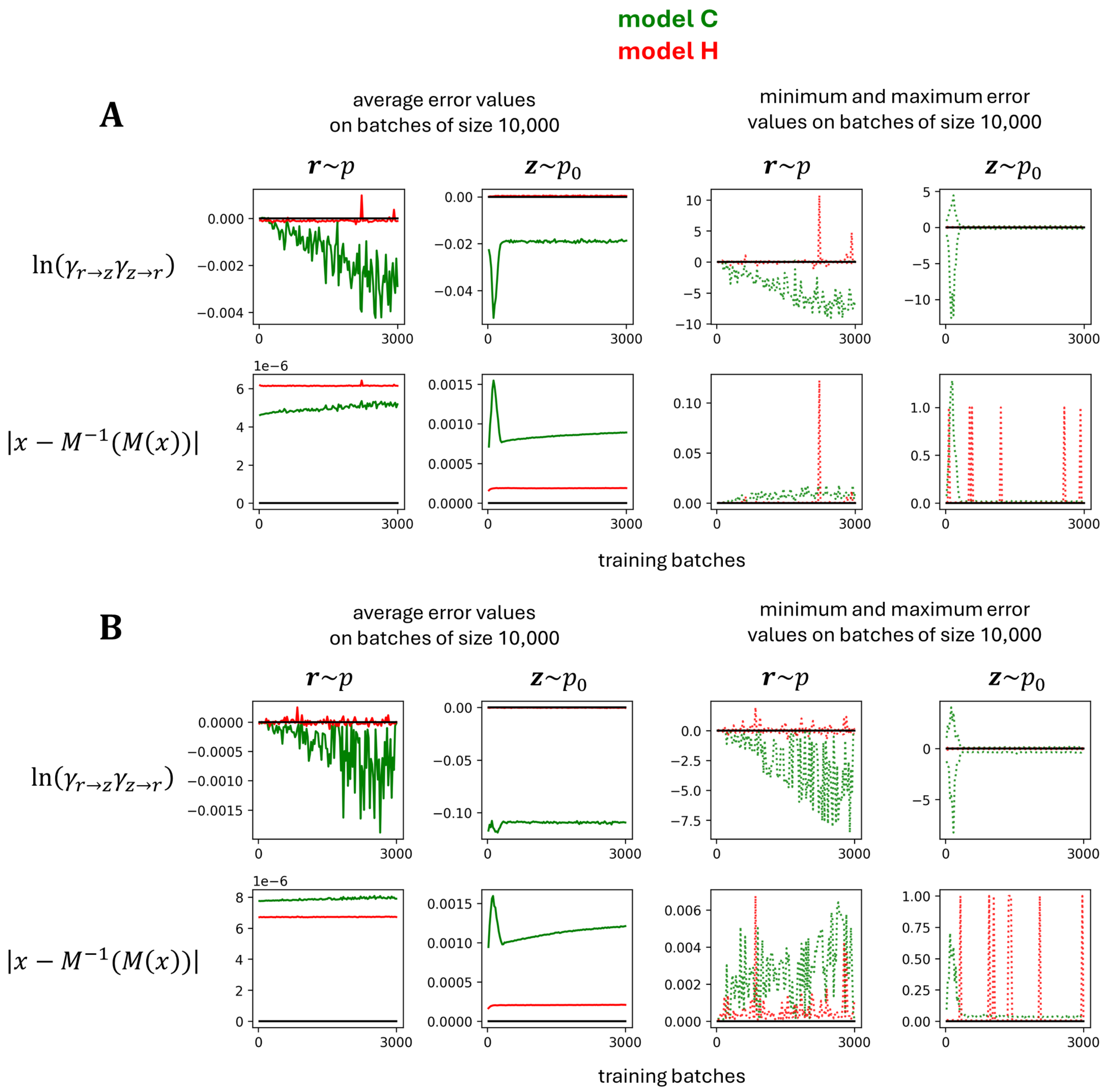

Figure S7: Analysis of the invertibility errors in the two types of PGM architectures during training on random 3,000 training batches. Two training datasets were used: (A) Ic 221 (32 molecules) at $T = 50C$, (B) XI 212 (32 molecules) at $T = 50C$. The quantities plotted describe the mean (solid coloured lines), maximum and minimum (dotted coloured lines) deviations from zeros error (solid black), in the two types of quantities: $\ln(\gamma_{r\to z}) + \ln(\gamma_{z\to r})$ (i.e., the difference in log of the infinitesimal volume change between the forward and inverse mapping of the same set of data-points) and $|x - \mathcal{M}^{-1}(\mathcal{M}(x))|$ (i.e., the absolute error between samples that were mapped forward and then backwards). Samples drawn from the base distribution are denoted by $\boldsymbol{z} \sim p_0$, and samples drawn from the MD validation set are denoted with $\boldsymbol{r} \sim p$. Both quantities should be zero in the absence of numerical errors in the mapping.
